# Is any job better than no job at all? Studying the relations between employment types, unemployment and subjective health in Belgium

**DOI:** 10.1186/s13690-017-0225-5

**Published:** 2017-08-24

**Authors:** Karen Van Aerden, Sylvie Gadeyne, Christophe Vanroelen

**Affiliations:** Interface Demography, Pleinlaan 2, 1050 Brussels, Belgium

**Keywords:** Unemployment, Employment quality, General health, Mental health, Household situation, Social support

## Abstract

**Background:**

This study focuses on the health impact of the labour market position, since recent research indicates that exposure to both unemployment and precarious employment causes serious harm to people’s health and well-being. An overview of general and mental health associations of different labour market positions in Belgium is provided. A distinction is made between employment and unemployment and in addition between different types of jobs among the employed, taking into account the quality of employment. Given the fact that precarious labour market positions tend to coincide with a precarious social environment, the latter is taken into consideration by including the composition and material living conditions of the household and the presence of social support.

**Methods:**

Belgian data from the 1st Generations and Gender Survey are used. A Latent Class Cluster Analysis is performed to construct a typology of labour market positions that includes four different types of waged employment: standard jobs, instrumental jobs, precarious jobs and portfolio jobs, as well as self-employment and unemployment. Then, binary logistic regression analyses are performed in order to relate this typology to health, controlling for household situation and social support. Two health outcomes are included: self-perceived general health (good versus fair/bad) and self-rated mental health (good versus bad, based on 7 items from the Center for Epidemiologic Studies Depression Scale).

**Results:**

Two labour market positions are consistently related to poor general and mental health in Belgium: unemployment and the precarious job type. The rather small gap in general and mental health between both labour market positions emphasises the importance of employment quality for the health and well-being of individuals in waged employment. Controlling for the household level context and social support illustrates that part of the reported health associations can be explained by the precarious social environment of individuals in unfavourable labour market positions.

**Conclusions:**

The results from this study confirm that the labour market position and social environment of individuals are important health determinants in Belgium.

## Background

In Western capitalist societies, the quality of employment conditions and relations has changed considerably in recent decades due to an extensive focus on labour market competitiveness and flexibility [1]. Also in Belgium, the transformation from a Fordist society based on industrial mass production to a Post-Fordist service economy went hand in hand with an erosion of the previously dominant, highly regulated model of full-time, lifelong employment in a fixed organisation [2, 3]. Apart from the growth of non-standard or atypical forms of work, employment also became more flexible in terms of working hours, opportunities for career progression and employment relations, thus altering various aspects of the employment experience [4]. Although an increasing body of recent literature provides evidence on the potential health effects of flexible or atypical employment forms, few researchers have used a multidimensional approach to study the health effects of employment quality [5]. Research including several employment quality features at once does find clear relations with employees’ health and well-being. Scott-Marshall & Tompa have studied the health consequences of precarious employment experiences by simultaneously including several key aspects of work-related precariousness (contract type, working hours, union coverage, income and benefits, supervisory responsibility and risk of exposure to physical hazards) in logistic regression analyses. They show that exposure to these aspects results in adverse consequences for general and functional health [6]. Research also used multidimensional scales to operationalise employment quality, such as the Employment Precariousness Scale (EPRES). This scale contains six dimensions: temporariness, disempowerment, vulnerability, wages, rights and exercising rights. A study using the EPRES shows that employment precariousness is related to poor mental health among Spanish employees [7]. Another example of a multidimensional measurement approach towards the quality of employment consists of the construction of job types based on information about key employment features. A recent study based on this approach shows clear associations between five ideal-typical employment arrangements on the one hand and self-perceived mental and general health on the other hand [8]. Adverse health outcomes are more often reported by employees holding a ‘precarious’ job, illustrating that precarious employment situations – characterised by the accumulation of ‘low quality’ job features – are an important determinant of health and well-being [8–10].

At the same time, individuals who did not succeed in finding a job – and experience unemployment as a result of that – also show lower scores for health and well-being indicators [11]. Thanks to a longstanding and extensive body of research, the health effects of exposure to unemployment are exceptionally well documented and explained. Jahoda was one of the first authors to write about the importance of employment for people’s health [12]. Warr also wrote a thorough review of the health effects of both (paid) employment and unemployment. He concludes that – while several characteristics of employment are potentially damaging to health – transitions from employment into unemployment are generally accompanied by deteriorating health outcomes for the persons involved [13]. Generally, recent studies confirmed these harmful effects of unemployment for both mental [14–16] and general health [17, 18], but some studies relate this to a health selection effect and thus doubt the causal link from unemployment to poor health [19]. Other studies have demonstrated the positive health effects of (re-)employment, while focussing at the same time on the observation that poor quality jobs are associated with worse health than good quality jobs [20].

Employment quality and unemployment are rarely investigated simultaneously since most studies either compare the health status of the unemployed versus the employed or examine the health status of employed individuals. Studies that do include both aspects tend to use the stability of employment as a benchmark to differentiate between types of jobs [21, 22]. Those studies do not entirely grasp the fact that the combination of several employment features determines the quality of a job. This article aims to fill this gap by providing an overview of general and mental health associations of labour market positions in Belgium, differentiating between employment and unemployment, but also between several types of jobs among the employed. Information on five dimensions representing the quality of employment conditions (employment stability, material rewards, workers’ rights and social protection, working time arrangements and employability opportunities) will be used to identify different types of waged employment in Belgium, using a latent class approach [8, 23].

The individual labour market situation tells only part of the story. Its health impact depends on the wider social context individuals are embedded in [24]. Precarious job features such as a low income or flexible/irregular working hours are likely to have a different impact depending on the household situation of the worker involved [24, 25]. Following this reasoning, unemployment or precarious types of employment could be assumed to lead to more (health) problems for those individuals (with dependents) living in a household without other income-earners or in a household combining multiple precarious jobs. Vosko uses the term “precarious households” to describe this phenomenon [26]. The observation that precarious labour market positions tend to coincide with precarious living arrangements compels the need to study the ‘net health associations’ of labour market positions [24].

The aim of this article is thus twofold. In first instance, it seeks to examine the relations between labour market position (type of employment or unemployment) and general and mental health in Belgium. In second instance, this study aims to investigate the role of the household context and social support in the relation between labour market position and health.

## Methods

### Data

The analyses presented are based on the first wave of the Belgian Generations & Gender Survey (GGS), conducted between February 2008 and May 2010. Of the 17,836 individuals that were selected using the National Register, 7163 participated in the survey. The GGS covers a large number of topics, including detailed information on the labour market position, household situation and general and mental health of the respondents. Considering the objectives of this article, the analyses will be limited to the working-age population (aged 18–64 at baseline) and to those respondents in (self-)employment or unemployed but looking for a job. Finally, 4377 respondents were included in the analyses. The characteristics of this sample are shown in Table 1.

**Table 1:** Characteristics of the sample

	n	%
Labour market position		
Employee	3443	78.7
Self-employed	523	11.9
Unemployed	411	9.4
Sex		
Male	2230	50.9
Female	2147	49.1
Age		
18–29	871	19.9
30–49	2450	56.0
50–64	1056	24.1
Household composition		
Working partner	2771	64.8
Non-working partner	684	16.0
No partner	822	19.2
Home ownership		
Owner	3206	73.5
Renter	1154	26.5
Financial situation		
Able to make ends meet and save	2609	60.1
Able to make ends meet, unable to save	416	9.6
Difficult to make ends meet	1315	30.3
Social support		
High	3457	79.3
Low	900	20.7
Self-perceived general health		
Fair/bad/very bad	596	13.6
Good/very good	3781	86.4
Self-rated mental health		
Poor	484	11.1
Good	3881	88.9

Source: Generations & Gender Survey Belgium

### Constructing a labour market typology

The main independent variable is the individual labour market position. Three groups of respondents are identified, based on their main activity status: employees, self-employed and unemployed. The group of employees is classified according to the quality of their job. For this purpose, a typology of jobs was constructed by means of a Latent Class Cluster Analysis (LCCA), using Latent Gold 4.5™ software. LCCA is a non-parametric alternative for Structural Equation Modelling (SEM) that uses the distribution of a set of manifest indicators in the sample to classify respondents in a limited number of latent categories. This results in the construction of an empirical typology, based on the degree of similarity of respondents regarding these indicators [23]. Nine employment conditions indicators are included in the LCCA, using sample weights. Missing data were directly modelled in the likelihood function of the model, assuming missing at random (MAR). This means that the classification of the respondent under consideration is based on the respondent’s other observed characteristics in the model.

The first employment conditions indicator included in the LCCA is *type of employment contract.* This indicator distinguishes between permanent and temporary contracts. The second indicator provides information on the *average monthly net amount of income* from the main job, subdivided into four categories: less than €1000, €1000–€1499, €1500–€2499 or more than €2499. The third indicator shows how many of the following *non-wage benefits* the respondent is entitled to: childcare; healthcare or medical insurance; housing; meal vouchers; a company car that can be used for private purposes and pension savings through a group insurance. The fourth indicator reveals whether or not the employer allows *flexible working times for personal reasons*. The fifth indicator, *exceptional working times*, shows if the respondent is confronted with working times that can be considered ‘unsocial’, such as evenings, nights, weekends, shift work or multiple working periods per day. The sixth indicator separates full-time and voluntary part-time from *involuntary part-time employment*. Part-time employment is considered involuntary in nature if it occurs because no full-time employment was found or because it was imposed by the employer. Indicator seven focuses on the presence of *long working hours*: working 40 h or less, between 40 and 48 h or more than 48 h per week. The eighth indicator shows whether respondents are confronted with *irregular working times*: working on call, according to an irregular work schedule or any other form. The final indicator considers *training opportunities*: did the respondent receive free or subsidized education/training in the context of his/her job? The overall prevalence for these nine indicators is shown in the first column of Table 2.

**Table 2:** Distribution of cluster probabilities over employment conditions indicators

	Overall prevalence	Standard jobs	Instrumental jobs	Precarious jobs	Portfolio jobs
Cluster size		0.3815	0.3267	0.1636	0.1281
Type of employment contract					
Permanent	0.9060	0.9582	0.9044	0.7269	0.9807
Temporary	0.0940	0.0418	0.0956	0.2731	0.0193
Average monthly net income					
Less than €1000	0.0955	0.0421	0.0004	0.4821	0.0088
€1000 - €1499	0.3278	0.2688	0.4284	0.5012	0.0324
€1500 - €2499	0.4747	0.5841	0.5529	0.0165	0.5279
€2500 or more	0.1020	0.1050	0.0183	0.0002	0.4309
Non-wage benefits					
0	0.2972	0.0862	0.4566	0.6326	0.0944
1	0.2391	0.1877	0.3262	0.2419	0.1656
2	0.2115	0.3005	0.1601	0.0940	0.2271
3 or more	0.2522	0.4257	0.0571	0.0316	0.5129
Flexible working times for personal reasons					
No	0.4178	0.3141	0.5973	0.4309	0.2544
Yes	0.5822	0.6859	0.4027	0.5691	0.7456
Exceptional working times					
No	0.7715	0.7744	0.8106	0.7443	0.6978
Yes	0.2285	0.2256	0.1894	0.2557	0.3022
Involuntary part-time employment					
FT & voluntary PT	0.9462	0.9946	0.9866	0.7098	1.0000
Involuntary PT	0.0538	0.0054	0.0134	0.2902	0.0000
Long working hours					
40 h or less	0.8081	0.9392	0.8620	0.9890	0.0469
40 h – 48 h	0.0942	0.0606	0.0657	0.0067	0.3791
More than 48 h	0.0978	0.0002	0.0723	0.0043	0.5741
Irregular working times					
No	0.9454	0.9547	0.9511	0.9450	0.9036
Yes	0.0546	0.0453	0.0489	0.0550	0.0964
Training opportunities					
No	0.3504	0.0944	0.5582	0.6977	0.1414
Yes	0.6496	0.9056	0.4418	0.3023	0.8586

Source: Generations & Gender Survey Belgium

The best-fitting typology of jobs is obtained by extending the number of clusters stepwise. In first instance, three formal model fit indicators (the Akaike Information Criterion, Bayesian Information Criterion and Consistent Akaike Information Criterion) are examined in order to identify the most parsimonious model. The lower their values, the better the estimated model fits the observed pattern of relations in the sample [27]. Of course, the theoretical meaning of the measurement model is also taken into account. Therefore, in second instance, interpretation of the relations between job types and employment conditions indicators helps to decide on the final number of clusters [23]. The relations between manifest indicators and latent clusters are reflected in conditional probabilities (presented in Table 2). Both the model fit criteria and the conditional probabilities indicate that a model with four clusters corresponds best with the observed pattern of relations in the data.

Each of the four job types is given a name based on their characteristic employment conditions. The first group (38.1%) contains jobs that are characterized by overall beneficial employment conditions. These jobs are described as “*standard jobs*” because they are the largest category and because of their resemblance to the standard model of employment in the Fordist period of industrial mass production [23]. The second category (32.7%) contains jobs that are called “*instrumental jobs*” because – based on the profile of manifest indicators – they can be conceived as an instrumental transaction between worker and employer. They are relatively stable, full-time or voluntary part-time jobs that provide a sustainable income for work with relatively good working hours but with few extra advantages (non-wage benefits, training and being allowed flexible working times for personal reasons). The third cluster (16.4%) is distinguished by overall adverse features, but specifically by high probabilities of instability, low income and involuntary part-time employment. These jobs are therefore described as “*precarious jobs”*. The fourth cluster (12.8%) contains jobs with overall beneficial employment conditions, except for the high probabilities of irregular, exceptional and (very) long working hours. These jobs are called “*portfolio jobs*”, because they are marked by strenuous working times, but also by high probabilities for contract stability, high income levels and receiving multiple non-wage benefits.

The final labour market typology contains six categories: five types of employment and unemployment. This means that two groups of respondents are added to the standard, instrumental, precarious and portfolio jobs: the self-employed and the unemployed. They are included in the typology as binary variables, with value 0 assigned to those individuals who are not in respectively self-employment/unemployment and value 1 assigned to those who are self-employed/unemployed.

### Household-level context and social support

Three indicators representing respondents’ household situation are introduced as control variables in the analyses. The first indicator sheds light on the *household composition* by distinguishing between respondents without a partner, with an unemployed partner and with an employed partner. Two indicators are retained to reflect the *household’s material living conditions*. The first one distinguishes owners and renters. The second indicator provides information on the household’s self-perceived financial situation, indicating whether the total monthly income allows to make ends meet and to save money (under normal circumstances). Three categories are discerned: able to make ends meet and save money, able to make ends meet but unable to save money and unable to make ends meet. A third indicator representing the household’s material living conditions, material deprivation, was left out of the final analyses because of the correlation with the financial situation variable. Including both indicators simultaneously resulted in a model almost identical to the model presented here (with financial situation but without material deprivation). *Social support* is operationalized using the following six items from the De Jong Gierveld loneliness scale: (1) There are plenty of people that I can lean on in case of trouble, (2) I experience a general sense of emptiness, (3) I miss having people around, (4) There are many people that I can count on completely, (5) Often, I feel rejected and (6) There are enough people that I feel close to [28]. First, items 2, 3 and 5 were recoded so that all items are scored in the same direction. Then, the six items were summed and the resulting scale was transformed in a scale ranging from 0 until 10. A cut-off value of 7 is applied, because it allows to isolate the lowest fifth of the social support distribution from the rest of the sample.

### Self-perceived general and mental health

The first outcome included in this study, *self-perceived general health*, is measured using the question “How is your health in general?”. The five possible responses (very good, good, fair, bad, very bad) are recoded to create an outcome that distinguishes respondents in (very) good general health from those in fair or (very) bad general health. The second outcome, *self-rated mental health*, is measured using information on how often respondents (1) felt they could not shake of the blues, (2) felt depressed, (3) thought their live had been a failure, (4) felt fearful, (5) felt lonely, (6) had crying spells, (7) felt sad in the previous week. These seven items are used because they are the only items of the 20-item “Center for Epidemiologic Studies Depression Scale” available in the GGS [29]. In our study, the established cut-off value between good and poor mental health (16/60) was converted to match the seven items available in our data (5.6/21).

### Methods of analysis

To examine the associations between the labour market typology and self-perceived general and mental health binary logistic regression analysis is applied, using IBM SPSS Statistics 22™ software. Data are weighted using sample weights. For the typology of jobs, the latent class probabilities per respondent are included as independent variables. This means that each respondent’s probability to belong to a certain job type is expressed in a decimal value between 0 and 1. Each respondent in waged employment thus receives four scores (one for each job type) that add to 1. The main advantage of this approach (compared to modal assignment, where respondents are exclusively assigned to a single cluster) is that classification errors are minimised because latent class probabilities include the information regarding the uncertainty of classifying cases in a particular category of the typology [27]. Self-employment and unemployment are added to the typology as dummy variables: value 1 (as opposed to value 0) means that the respondent is respectively self-employed or unemployed.

Tables 3 and 4 present the odds ratios (ORs) resulting from the analyses with respectively poor general health and poor mental health as dependent variables. The introduction of the labour market typology as a set of variables ranging from 0 to 1 means that reported effects should be interpreted as maximum effect sizes, corresponding with a 100% resemblance to the labour market position under consideration. The standard job type is used as reference category in the analyses. The ORs thus describe the odds of occurrence of poor general or mental health given full exposure to a particular labour market position (e.g. being self-employed or a 100% resemblance with the portfolio job type), compared to the odds of these outcomes occurring in the absence of that exposure (e.g. a 100% resemblance with the standard job type). Four models are shown per outcome: a basic model, a model additionally controlled for sex and age, a model additionally controlled for three household situation variables and a final model additionally controlled for social support.

**Table 3:** Relations between labour market position and poor general health

	Basic model	Model 1	Model 2	Model 3
Standard jobs	Ref.	Ref.	Ref.	Ref.
Instrumental jobs	1.15 n.s. *(0.78–1.69)*	1.27 n.s. *(0.86–1.88)*	1.07 n.s. *(0.72–1.60)*	0.98 n.s. *(0.65–1.47)*
Precarious jobs	2.07 *** *(1.44–2.95)*	2.43 *** *(1.67–3.52)*	1.70 ** *(1.15–2.49)*	1.53 * *(1.04–2.26)*
Portfolio jobs	0.70 n.s. *(0.44–1.13)*	0.72 n.s. *(0.44–1.17)*	0.79 n.s. *(0.48–1.29)*	0.82 n.s. *(0.50–1.33)*
Self-employment	1.41 * *(1.00–1.98)*	1.37 n.s. *(0.97–1.93)*	1.35 n.s. *(0.96–1.91)*	1.29 n.s. *(0.91–1.84)*
Unemployment	3.15 *** *(2.28–4.34)*	3.54 *** *(2.54–4.92)*	2.09 *** *(1.47–2.97)*	1.85 ** *(1.29–2.65)*
Women (men = ref.)		1.09 n.s. *(0.90–1.32)*	1.14 n.s. *(0.93–1.38)*	1.16 n.s. *(0.95–1.42)*
Age (50–64 = ref.)		***	***	***
18–29		0.25 *** *(0.18–0.34)*	0.24 *** *(0.17–0.33)*	0.26 *** *(0.18–0.36)*
30–49		0.63 *** *(0.52–0.77)*	0.60 *** *(0.49–0.74)*	0.60 *** *(0.49–0.75)*
HC (working partner = ref.)			n.s.	n.s.
Non-working partner			1.22 n.s. (0.94–1.57)	1.20 n.s. *(0.93–1.55)*
No partner			1.19 n.s. (0.93–1.52)	1.08 n.s. *(0.84–1.39)*
Renter (owner = ref.)			1.30 * (1.05–1.61)	1.26 * *(1.01–1.56)*
FS (able to make ends meet and save = ref.)			***	***
Able to make ends meet, unable to save			1.23 n.s. *(0.89–1.71)*	1.13 n.s. *(0.81–1.58)*
Difficult to make ends meet			2.21 *** *(1.78–2.74)*	1.89 *** *(1.51–2.36)*
Low social support (high = ref.)				2.49 *** *(2.03–3.05)*
Constant	0.11 ***	0.16 ***	0.12 ***	0.11 ***
Nagelkerke R Square	0.034	0.071	0.104	0.134

95% Confidence intervals are shown in parentheses

Model 1: controlled for sex and age

Model 2: controlled for sex, age and household situation (HC: household composition, home ownership and FS: financial situation)

Model 3: final model controlled for sex, age, household situation and social support

n.s. *p* > 0.05, * *p* < 0.05, ** *p* < 0.01,*** *p* < 0.001

Source: Generations & Gender Survey Belgium ( *n* = 4208)

**Table 4:** Relations between labour market position and poor mental health

	Basic model	Model 1	Model 2	Model 3
Standard jobs	Ref.	Ref.	Ref.	Ref.
Instrumental jobs	2.34 *** *(1.49–3.65)*	2.56 *** *(1.62–4.03)*	2.07 ** *(1.30–3.31)*	1.76 * *(1.08–2.88)*
Precarious jobs	4.06 *** *(2.72–6.07)*	3.50 *** *(2.32–5.29)*	2.15 *** *(1.40–3.31)*	1.74 * *(1.10–2.75)*
Portfolio jobs	0.75 n.s. *(0.41–1.39)*	1.00 n.s. *(0.54–1.87)*	1.26 n.s. *(0.66–2.39)*	1.46 n.s. *(0.75–2.83)*
Self-employment	1.16 n.s. *(0.73–1.83)*	1.32 n.s. *(0.83–2.10)*	1.25 n.s. *(0.78–2.01)*	1.14 n.s. *(0.69–1.88)*
Unemployment	6.70 *** *(4.66–9.63)*	6.89 *** *(4.75–9.99)*	3.29 *** *(2.21–4.89)*	2.70 *** *(1.77–4.13)*
Women (men = ref.)		1.93 *** *(1.55–2.41)*	2.04 *** *(1.62–2.56)*	2.42 *** *(1.89–3.09)*
Age (50–64 = ref.)		n.s.	n.s.	n.s.
18–29		0.77 n.s. *(0.57–1.05)*	0.72 * *(0.53–1.00)*	0.92 n.s. *(0.65–1.30)*
30–49		0.92 n.s. *(0.72–1.18)*	0.86 n.s. *(0.66–1.12)*	0.89 n.s. *(0.67–1.17)*
HC (working partner = ref.)			***	***
Non-working partner			1.07 n.s. *(0.78–1.46)*	1.05 n.s. *(0.75–1.46)*
No partner			2.13 *** *(1.66–2.74)*	1.98 *** *(1.51–2.58)*
Renter (owner = ref.)			1.21 n.s. *(0.96–1.53)*	1.11 n.s. *(0.86–1.42)*
FS (able to make ends meet and save = ref.)			***	***
Able to make ends meet, unable to save			2.17 *** *(1.51–3.11)*	1.89 *** *(1.29–2.77)*
Difficult to make ends meet			3.45 *** *(2.69–4.43)*	2.62 *** *(2.01–3.42)*
Social support				7.44 *** *(5.92–9.36)*
Constant	0.06 ***	0.04 ***	0.02 ***	0.01 ***
Nagelkerke R Square	0.081	0.099	0.181	0.311

95% Confidence intervals are shown in parentheses

Model 1: controlled for sex and age

Model 2: controlled for sex, age and household situation (HC: household composition, home ownership and FS: financial situation)

Model 3: final model controlled for sex, age, household situation and social support

n.s. *p* > 0.05, * *p* < 0.05, ** *p* < 0.01,*** *p* < 0.001

Source: Generations & Gender Survey Belgium ( *n* = 4201)

## Results

Results for the relations between the labour market typology and poor general health are shown in Table 3. The results from the first model – controlled for sex and age – show that unemployment and precarious jobs are associated with significantly higher odds to report poor general health than the standard job type. The introduction of the three household situation indicators in model 2 lowers the odds for most labour market positions, but the differences with the standard job type remain statistically significant for both the unemployed and the precarious job type. The odds ratios observed in model 1 are strongly reduced in the final model (additionally controlled for social support), but remain significant for both the unemployed and respondents holding a job resembling the precarious type. Home ownership, financial situation and social support are significantly related to self-perceived general health. The household context and social support indicators seem to be particularly important for the general health level of respondents in unemployment.

Table 4 presents the results for the relations between the labour market typology and poor self-rated mental health. In model 1, unemployment, the precarious job type and the instrumental job type show significantly higher odds than the standard job type to report poor mental health. The difference is largest for the unemployed, followed by the respondents holding a job that resembles the precarious type and those holding a job resembling the instrumental type. Controlling for the household context (model 2) and social support (model 3) generally lowers the odds of poor mental health for the different labour market positions, but differences remain significant for the three labour market positions. Household composition, financial situation and social support are significantly related to self-rated mental health. The household situation and the presence of social support appear to be very important for the unemployed and to a lesser extent for respondents holding a job resembling the precarious type.

## Discussion

Two labour market positions are consistently related to poor self-perceived general and mental health in Belgium: unemployment and the precarious job type. The instrumental job type is related to poor self-rated mental health only. The introduction of the individual’s household situation and the presence of social support provides us with important insights considering the relation between labour market position and health.

First of all, the reduction in odds ratios following the inclusion of the social context variables in the model is in accordance with previous research indicating that part of the health problems associated with unemployment and low-quality employment are due to the fact that these labour market positions often coincide with a precarious or deprived situation at the household level [25]. Research has illustrated the importance of employment at the household level during recent decades, amongst other things because of changing household structures [30, 31]. Giatti, Barreto & César report a relationship between unemployment and poor self-rated health at the individual level and an additional association between poor self-rated health and living in a household with at least one informal or unemployed worker [32]. However, Scutella & Wooden find no evidence for an additional mental health effect of living in a jobless household among unemployed individuals [33]. Lim, Kimm & Song examine the interaction between employment status and household income [34]. They conclude that the self-perceived health of precarious workers is more strongly affected by household income, due to the instability of their employment status.

The results of our analyses do not only point out that the accumulation of health-damaging societal positions is a worrisome reality for a part of the Belgian population. The rather small gap in poor general and mental health between the unemployed and individuals in precarious employment in the final model suggests that low-quality employment and unemployment have an equally strong link to health. This observation has been largely overlooked in studies merely focussing on the health associations of unemployment and opens up a debate about the importance of job quality in addition to merely promoting employment for the unemployed. Several other authors have also emphasised that any job is not necessarily better for health than no job at all [13, 20, 35, 36].

The use of latent classes based on multiple employment quality indicators to represent different types of waged employment is another innovative aspect of this study. It allows to broaden the mere comparison between employment and unemployment by taking into account the multidimensional nature of the employment situation. Previous research on data from the European Working Conditions Survey has applied the same technique for employees living in the EU27 [8, 23]. The job types that were discovered are strikingly similar, with that difference that two precarious job types (precarious intensive and precarious unsustainable employment) were found instead of one [23]. The fact that more or less the same job types were found in a different dataset and using a different set of variables is a strong validation of our approach to employment quality. The results from this European research also confirm the status of precarious employment as the most detrimental job type for employees’ general and mental health [8].

The results of this study show the importance of high-quality employment for individuals’ general and mental health situation and suggest that not just any job will do. However, further research using a multidimensional approach towards the quality of employment is needed to confirm the health gradient from unemployment over low-quality jobs to high-quality employment. So far, empirical evidence on this topic remains limited for Belgium as well as for other countries. In particular, longitudinal research designs including employment quality could provide plenty of extra information with regard to the health effects of labour market mobility and about the relative strength of both directions of causality. This would also allow to study whether the actual change from unemployment to a low-quality job results in an improvement in health or not.

The results of this study also suggest that the household situation and social support are important factors mediating the relationship between labour market position and health. However, the analyses in this study do not allow a formal test of individuals’ social context as a pathway linking labour market position to health. Further research is needed to clarify the relationship between labour market position, social context and health.

The limitations of this study are mainly related to our use of secondary data and the resulting restrictions with regard to the operationalisation of our main concepts: labour market position, household situation, social support and health. The cross-sectional nature of our data implies that no statements about the causality of the reported associations can be made. This also means that we cannot rule out the existence of a certain bias due to health selection effects. The existence of health selection entails that part of the relation between labour market position and health is shaped by the fact that individuals with lower health are less likely to obtain a (high-quality) job [37]. Finally, the limited number of cases in our dataset made it impossible to include more labour market positions in the typology or to perform gender-specific analyses. Future research is needed to clarify the health associations of those individuals in (early) retirement, housekeepers or students and to examine the role of gender in the relation between labour market position, social situation and health. However, the use of GGS-data in this study is also one of its innovative features, since data from this survey have been used mainly for research on fertility and family formation. The application of this dataset in research focussing on labour market position and health is quite unique and proves the value and the large scope of these data.

## Conclusion

This article presents evidence of clear relations between the labour market position and the self-perceived general and mental health of working-aged individuals in Belgium, even when taking into account the fact that an individual’s labour market position is necessarily embedded in a broader social context. The unemployed and those employed in low-quality (precarious) jobs are most likely to face health problems.

This paper makes several contributions to occupational health literature. While previous research has mainly focused on the health consequences of unemployment (often in comparison with the employed in general), this study pays attention to the existing diversity in the employment situations of workers. Not only self-employed are included, but on top of that an innovative approach is used to integrate information with regard to the quality of employment conditions for those in waged employment. Finally, this article acknowledges that precarious labour market positions tend to coincide with precarious living arrangements and a lack of social support, by taking into account the household context and social support when considering the health associations of the individual labour market situation.

## Abbreviations

EPRES: Employment Precariousness Scale
FT: Fulltime
GGS: Generations & Gender Survey
LCCA: Latent Class Cluster Analysis
MAR: Missing at random
n.s.: Not significant
OR: Odds ratio
PT: Parttime
SEM: Structural Equation Modelling
